# Exploring the moderating role of mindfulness, mindful eating, and self-compassion on the relationship between eating-disordered quality of life and orthorexia nervosa

**DOI:** 10.1007/s40519-023-01542-7

**Published:** 2023-02-20

**Authors:** Eliza Kalika, Misba Hussain, Helen Egan, Michail Mantzios

**Affiliations:** Department of Psychology, Birmingham City University, Curzon Building, Office C325, Birmingham, B4 7DE UK

**Keywords:** Orthorexia Nervosa, Mindfulness, Mindful eating, Self-compassion, Quality of life

## Abstract

Orthorexia nervosa (ON) is characterised by an obsessive focus on healthy eating, following restrictive dietary practices and dietary restrictions escalating over time. The aim of this study was to explore mindfulness, mindful eating, self-compassion and quality of life in a female population. Two hundred eighty-eight participants completed Orthorexia, Self-Compassion, Mindful eating, Mindfulness and Eating Disorder Quality of Life scales. The results indicated that there was a negative relationship between ON and mindfulness, self-compassion and mindful eating. Furthermore, the present study found a positive relationship between lower quality of life and ON, while findings indicated that self-compassion and the awareness facet of mindfulness moderated the relationship between ON and QOL. The present results contribute to a better understanding of orthorexic eating behaviours in a female population, and identify the moderating capacity of self-compassion and mindfulness. Further implications and future directions are discussed.

Level of evidence Level V, cross-sectional descriptive study.

## Introduction

Research has shown that engaging in restrictive rituals for healthy eating can lead to the development of Orthorexia Nervosa (ON) (e.g., [14, 18]. ON was defined as an obsessive fixation on healthy eating [11]. Individuals who display orthorexic tendencies place significant emphasis on food quality and purity and spend a significant amount of time planning and preparing healthy meals [48]. The imposition of extreme restrictions can result in malnourishment and medical complications [15, 28], as well as severely reduce the enjoyment of food [18].

In social climates where engaging in extreme dieting restrictions is normalised and endorsed, distinguish between ON and healthy eating can be challenging. Bratman [11] proposes that symptomology including obsessive thoughts, compulsive behaviours, self-punishment and extreme restrictions help to distinguish healthy eating from ON. Currently, there are no diagnostic criteria for ON in the classification system (American Psychological Association [APA], 2013), although some recent literature has proposed guidelines that meet consensus by researchers and clinicians [25], indicative of progress in the field to a universal definition and descriptive factors, which are to be translated to diagnostic tools and measurements through future research.

Currently, however, the lack of one universally accepted diagnostic tool contributes to widely varying prevalence rates between 1 and 82.7% (e.g., [16], Depa et al., 2017; [72, 75]. In addition, some researchers identified that the scales used in the past may have overestimated the prevalence of orthorexic eating behaviours (e.g., [73], adding to the immense difference in prevalence rates. The present study utilised a new assessment tool referred to as the Orthorexia Nervosa Inventory (ONI) [72] to measure orthorexia, which met consensus in more closely measuring orthorexia by developers of other scales and pioneers in the field of orthorexia [25], Niedzielski & Kazmierczak-Wojtas, 2021). This tool takes into consideration the preoccupation with healthy food, physical and psychosocial impairments and emotional distress factors, which are all central to the conceptualisation of ON [72]. Significantly, elements that are accounted for in measuring ON propose implications for quality of life, especially when considered in parallel literature that has looked into disordered eating.

Quality of life (QoL) is defined as a patient-centred method of appraising the impact of symptoms on the individual’s health [32]. Individuals with AN, Bulimia Nervosa (BN) and Binge Eating Disorder (BED) have reported worse QoL than the general population (e.g., Agh et al., 2016, [21, 22, 41, 61, 95]. Research has shown that people with ON also experience reduced QoL due to self-punishment following dietary violations (Koven & Arby, 2015), avoidance of social gatherings where food is involved leading to social isolation [28], depression and anxiety (Bosi et al., 2017). Individuals who have experienced an ED for several years may suffer from impairments in psychological, physical, and social aspects of life [41, 95], but no studies have examined the quality of life specific to eating disorders in individuals who display orthorexic symptoms. While targeting QoL directly is one option, another indirect method of potentially enhancing QoL, and eating regulation that remains healthy and steers clear of orthorexia symptoms may be through mindfulness-based traits and interventions.

Mindfulness is defined as a psychological concept that involves consciously attending to external and internal experiences such as emotions, thoughts, and bodily sensations in a non-judgmental way [42]. Reviews of empirical research have indicated that mindfulness-based interventions (MBI) have improved psychological and physical well-being [20, 45]. Numerous studies also show that mindfulness has a positive effect on the quality of life, including in healthy individuals, patients with ulcerative colitis, asthma, multiple sclerosis, depression, and schizophrenia [39, 47, 78],Rayan & Ahman, 2016; [81]. One form of mindfulness, directed specifically towards the eating process is mindful eating. Mindful eating is defined as sustained attention to a sensory element of the eating experience (e.g., taste) and a non-judgmental (or non-evaluative) awareness of thoughts and feelings that are incongruent with the sensory elements of the present eating experience [50]. Within explorations of eating behaviours, mindful eating has been shown to challenge motivations. Mantzios et al. (2019a, b discovered that mindful eating interventions inspire a gradual shift from external motivations to internal motivations, which is linked to healthier eating behaviours [55, 57–59, 96]. Only one study has looked at mindful eating and self-compassion in relation to ON, and the findings suggested that mindful eating was not associated with ON, which was mostly attributed to the vegan population that was recruited and the naturally more restrictive diet, but self-compassion displayed a negative association with ON [43].

Another MBI that could be useful is founded in the theory of self-compassion, which is linked to mindfulness and mindful eating, and the relation to eating behaviours and food consumption [53, 54]. The understanding that suffering, inadequacy, and failure are all part of the human experience is descriptive as self-compassion [68]. Self-kindness, shared humanity, and awareness are the three components of this concept, and higher levels of self-compassion are linked to higher levels of happiness, life satisfaction and lower levels of shame, depression, and anxiety [69, 70]. Furthermore, Ferreira et al. (2013) have found that lower levels of self-compassion are associated with higher levels of body dissatisfaction, drive for thinness and eating disorder pathology. The nature of self-judgement can evoke distress, which could result in disordered eating, serving as a coping strategy for managing internal and external threats by avoiding criticism due to body shape and weight [35]. However, this can ultimately result in worse negative emotional states. As self-compassion addressed one’s thoughts, emotions and experiences with kindness and empathy, this can be used to regulate negative affect and threats (Meyer et al., 2018). A recent systematic review found evidence that self-compassion acts as a protective factor against body dysmorphia and eating disorders [12], whereas Adams and Leary (2007) found that utilising self-compassion intervention with restrictive eaters has reduced their distress-related eating. All these factors are associated with ON, such as restrictive eating [7] and self-judgement [19]. Therefore, utilising self-compassion may reduce the symptoms of ON, and improve the quality of life of an individual that displays orthorexic tendencies.

The first aim of this study was to explore mindfulness, self-compassion, mindful eating and orthorexia in the female population due to a greater prevalence of orthorexia in female samples (e.g., [23, 36], Parra Fernandez, 2018; [80]. The second aim of this study was to explore whether mindful eating, self-compassion and mindfulness moderate the relationship between ON and QoL. To our knowledge, only one study looked at mindfulness [84], and another study looked at mindful eating and self-compassion [43]. In accordance with previous literature, it is hypothesised that mindful eating, self-compassion and mindfulness will be negatively correlated with ON. Furthermore, the present study aimed to investigate eating disorder quality of life, which is based on the literature on quality of life in eating disorders and a novel investigation in the orthorexia literature, with a hypothesis that individuals with high orthorexic tendencies would demonstrate a lower quality of life. Additionally, the potential of mindfulness-based constructs moderating this relationship between orthorexia and QoL will be further explored.

## Methods

### Participants

The sample for the present study consisted of 288 female participants who were all adults (18 years or over; *M* = *24.79, SD* = *7.08*) with a mean Body Mass Index (BMI) of *M* = *24.26* kg/m^2^ (*SD* = *6.45)*. A total of 69.1% of participants identified as White, 19.8% as Asian, 3.5% as Black, 2.8% as Mixed and 4.9% as Other, Furthermore, the type of diet was also collected, the sample consisted of 75.4% of Omnivores, 22.2% of vegetarians and 2.4% as vegans. Participants were recruited through volunteering sampling by advertising the study on several social media platforms and forums such as Facebook, Instagram, Twitter, LinkedIn and MiniMins. The advertisement on Facebook has been posted in healthy eating groups requesting individuals to participate in the study. Individuals were also recruited through the university’s Research Participation Scheme. Those who participated in the scheme were rewarded with research credits upon completion of the study. Participants were informed via the information sheet that the inclusion criteria for this study required them to be over the age of 18, have good knowledge of the English language and not be diagnosed with an eating disorder.

### Materials

*Demographic information:* a set of question designed to collect general information about participants. Participants were required to report their age, gender, ethnicity, weight, height and type of diet e.g., vegan, semi-vegetarian, omnivore etc.

*Orthorexia Nervosa Inventory (ONI).* Scale developed by Oberle et al. [72]. It is a measure of ON symptomatology which includes 24 items assessing 3 factors of orthorexic behaviours such as impairments, behaviours and emotions. It utilises a 4-point Likert scale with the following responses: 1 (not at all true) to 4 (very true). The higher total score indicates greater severity of ON. Sample questions include “Just the thought of me eating something unhealthy makes me very anxious”. The Cronbach alpha for the present study was 0.947. Additionally, the Cronbach alpha was calculated for the subscales, impairments were 0.918, behaviours were 0.873 and emotions were 0.857.

*Five-Facet Mindfulness Questionnaire- Short Form (FFMQ).* This is a shorter version of the original 39-item FFMQ. This scale was developed by Baer et al. [5] and includes 15 items that measure five facets such as Observing, Describing, Acting with Awareness, Non-Judging of inner experience and non-Reactivity. This scale utilises a 5-point Likert scale with the following responses: 1 (never true) to 5 (always true). A score is combined for each facet of the scale. Sample questions include “I find myself doing things without paying attention”. The Cronbach alpha for the present study was 0.736. Additionally, the Cronbach alpha was calculated for the subscales, observing was 0.485, describing was 0.807, acting with awareness 0.714, non-judging of inner experience was 0.776 and non-reactivity was 0.637.

*The Mindful Eating Behaviour Scale (MEBS).* Scale developed by Winkens et al. [94], it contains 17 items that measures four components of mindful eating, these are focused eating, hunger and satiety cues, eating with awareness and eating without distraction. This scale utilizes a 5-point Likert scale with the following responses 1 (never) to 5 (always). The higher the score the more mindful the individual. It is recommended by the author to use the four subscales separately rather than producing a total score. Sample questions include “I notice how my food looks”. The Cronbach alpha for the present study was 0.790. Additionally, the Cronbach alpha was calculated for the subscales, eating while focusing on the food was 0.875, eating while paying attention to hunger and satiety cues was 0.894, being aware of eating was 0.907 and eating while not being distracted was 0.780.

*Self-Compassion Scale-Short Form (SCS-SF).* This is a shorter form of the original 26-item SCS, it was developed by Raes et al. [77] to measure self-compassion. The items are rated on a 5-point Likert scale with the following responses, 1 (never) to 5 (always). This scale includes three compassionate components and three unpassionate components, these components are self-kindness, self-judgement, common humanity, isolation, mindfulness, and over-identification. Sample questions include “I try to see my failings as part of the human condition”. The Cronbach alpha for the present study was 0.814.

*Eating Disorder Quality of Life (EDQOL).* Scale developed by Engel et al. [33] and it measures the impact of eating disorder symptoms on the individual’s quality of life. It includes 25-items which measure four domains: psychological, physical/cognitive, financial and school/work. Answers are given on a 4-point likert scale with the following responses, 0 (never) to 5 (always). The higher scores indicate the worse quality of life. Sample questions include “How often has your eating/weight made you feel lonely”. The Cronbach alpha for the present study was 0.951. Additionally, the Cronbach alpha was calculated for the subscales, psychological was 0.947, physical/cognitive was 0.911, financial was 0.931 and work/school was 0.949. Some studies explored the QoL in EDs using generic QoL measures such as SF-36 Health Survey (e.g., [40]. In the present study, the Eating Disorder Quality of Life measure (EDQOL) was used, which has been shown to be sensitive to the unique characteristics of ED. Engel et al. [32] indicated that QoL is an important element of ED assessment, and generic QoL measures needed to be adapted to account for the unique characteristics of ED.

### Procedure

Ethical approval was obtained from the ethical committee of an institution based in the midland region of the United Kingdom. Participants were recruited via forums and social media groups and were asked to share the study with their contacts. They were presented with information about the study such as inclusion and exclusion criteria, and the hyperlink to Qualtrics that directed them to the questionnaire. Furthermore, the university Research Participation Scheme was utilised where individuals gained research credits for participation. Participants were asked to read the information about the research in a Participant Information Sheet, which appeared prior to consenting. Participants consented and created a unique code to identify data in case of withdrawal. Participants were then presented with demographic information, ONI, FFMQ, MEBS, SCS-SF and EDQOL. After completion, the participant was presented with a debrief form that explained the aims of the study and the procedure in case of withdrawal. The study consisted of one online session, which lasted approximately 30 min.

### Data analysis

Prior to conducting the analysis of the data, assumptions were tested, despite bootstrapping and heteroscedastisity-consistent inference options eliminating the need for normality and homoscedasticity (e.g., [76]. First, the data were checked for outliers. Cook’s distance was used and the range was between 0 and 0.115 which indicated that there were no outliers. According to Hair et al. [37] the values between 2 and − 2 for Skewness and 7 to − 7 for Kurtosis are considered to be normal. The assumptions for normality were examined using Skewness and Kurtosis. Skewness scores for ONI, EDQOL, SCS, FFMQ and MEBS were 1.5, 0.94, 0.16, − 0.08 and − 0.16. Kurtosis scores for ONI, EDQOL, SCS, FFMQ and MEBS were 2.3, 0.29, 0.16, 0.88 and − 0.22. So, the data met the assumption for normality. Multicollinearity was tested using the variance inflation factor (VIF) values, the highest value was 1.5 which is below the value of 5 [87] meeting the assumption. Additionally, P-P plots and residual scatter plots supported linearity and homoscedasticity assumptions. Data analysis was conducted using SPSS software (version 25.0, IBM Corp., 2017. Pearson’s bivariate correlations were conducted to assess the associations between Orthorexia (ONI, Mindfulness (FFMQ, Mindful Eating (MEBS, Self-compassion (SCS and Eating Disorder Quality of Life (EDQOL (see Table 1). A further correlation analysis was conducted to examine the relationships between ONI, subscales of FFMQ and EDQOL (see Table 2).

**Table 1:** Bivariate correlations between BMI, ONI, FFMQ, MEBS, SCS-SF and EDQOL and descriptive statistics

	1	2	3	4	5	6	7	8	*M*	SD
(1) ONI									36.25	11.96
(2)BMI	0.056								24.26	6.45
(3) FFMQ	− 0.155*	− 0.006							45.32	7.02
(4) MEBS- Focused Eating	− 0.134*	0.002	0.183**						18.64	4.37
(5) MEBS- Hunger & Satiety	− 0.243**	− 0.197**	0.175**	0.452*					16.61	4.86
(6) MEBS- Eating with Awareness	− 0.179**	− 0.110	0.289**	0.214**	0.237**				10.93	3.36
(7)MEBS- Eating without distraction	− 0.084	0.012	0.296**	0.058	0.124*	0.508**			12.75	3.48
(8) SCS-SF	− 0.160**	− 0.055	0.548*	0.078	0.146*	0.131*	0.180**		2.79	0.63
(9) EDQOL	0.605**	0.123*	− 0.203**	− 0.233**	− 0.355**	− 0.360	− 0.244**	− 0.208**	3.78	2.90

*ONI* Orthorexia Nervosa Inventory, *BMI* Body Mass Index, *FFMQ* Five-Facet Mindfulness Questionnaire, *EDQOL* Eating Disorder Quality of Life Scale, *SCS-SF* Self-Compassion Scale-Short Form, *MEBS* Mindful Eating Behaviour Scale

^*^Correlation is significant at the .05 level

^**^Correlation is significant at the .01 level

**Table 2:** Bivariate correlations between subscales of ONI, SCS, FFMQ, MEBS and EDQOL and descriptive statistics

	1	2	3	4	5	6	7	*M*	SD
(1) Impairments (ONI)								14.15	5.06
(2) Behaviour (ONI)	0.828**							14.18	4.75
(3) Emotion (ONI)	0.657**	0.651**						8.55	3.47
(4) EDQOL	0.604**	0.461**	0.499**					3.78	2.90
(5) SCS-SF	− 0.116	− 0.038	− 0.274**	− 0.208**				2.79	0.63
(6) MEBS	− 0.183**	− 0.097	− 0.342**	− 0.442**	0.199**			59.15	10.82
(7)FFMQ	− 0.118	− 0.007	− 0.276**	− 0.203**	0.548**	0.345**		45.33	7.03

*ONI* Orthorexia Nervosa Inventory, *FFMQ* Five-Facet Mindfulness Questionnaire, *EDQOL* Eating Disorder Quality of Life Scale, *SCS-SF* Self-Compassion Scale-Short Form, *MEBS* Mindful Eating Behaviour Scale

^*^Correlation is significant at the .05 level

^**^Correlation is significant at the .01 level

Furthermore, moderation analysis was used to determine which variables moderated the relationship between orthorexia nervosa on quality of life in the female sample. Hayes’ [76] PROCESS macro (v3.3) was installed on SPSS (version 25.0) and was used to conduct moderation analyses (model 1). No covariates were controlled for this moderation model. Participant’s BMI has been calculated using the height and weight information provided by the participant.

## Results

A multiple correlation analysis has been used to identify which scales (BMI, FFMQ, MEBS, SCS-SF, EDQOL) relate to ONI.

Inter-correlations between BMI, ONI, FFMQ, MEBS, SCS-SF and EDQOL, are presented in Table 1. Findings indicate that there is a significant negative relationship between ONI and Focused eating MEBS (*p* = 0.029), Hunger and Satiety MEBS (*p* < 0.001), Eating Awareness MEBS (*p* = 0.004), FFMQ (*p* = 0.012) and SCS (*p* = 0.008). The only positive relationship was EDQOL (*p* < 0.001), Only eating without Distraction subscale in MEBS was not significant as well as BMI. In addition, correlational analysis on the subscales of ONI was performed results are presented in Table 2.

A further correlation analysis has been conducted between the ONI, and subscales of FFMQ, and EDQOL, the findings are presented in Table 3. For FFMQ, it showed negative associations with the Awareness subscale (*p* < 0.001) and non-judging subscale (*p* < 0.001). All other associations were non-significant for the FFMQ scale. Finally, the last scale of EDQOL showed all positive associations with Psychological, (*p* < 0.001), Cognitive (*p* < 0.001), Financial (*p* < 0.001) and Work subscales (*p* < 0.001).

**Table 3:** Bivariate correlations between ONI and subscales of FFMQ and EDQOL

	EDQOL- Psychological	EDQOL- Cognitive	EDQOL- Financial	EDQOL- Work	FFMQ- Observing	FFMQ-Describe	FFMQ-Awareness	FFMQ- Non-Judging	FFMQ- non-reactivity
ONI	0.481**	0.548**	0.451**	0.439**	0.102	− 0.056	− 0.210**	− 0.335**	0.080

*ONI* Orthorexia Nervosa Inventory, *FFMQ* Five-Facet Mindfulness Questionnaire, *EDQOL* Eating Disorder Quality of Life Scale

^*^Correlation is significant at the .05 level

^**^Correlation is significant at the .01 level

The analysis also ran multiple moderation model analyses. The first moderation model analysis used ONI as the independent variable, EDQOL as a dependent variable, and SCS as a potential moderator (see Table 4). The ONI significantly predicted the EDQOL *b* = 0.15, *p* < 0.001, 95% Cl [0.13, 0.18], whereas SCS did not *b* = − 0.38, *p* > 0.05, 95% CI − 0.84, 0.08]. However, there was a significant ONI x EDQOL *b* = 0.08, *p* = 0.0027, 95% CI [0.03, 0.13] indicating that there is evidence of SCS acting as a moderator. This has been explored with the use of simple slopes around the EDQOL mean, at the -1SD there was a significant relationship between ONI and SCS *b* = 0.11, *p* < 0.001, 95% CI [0.07, 0.14], while this remained significant at the EDQOL mean *b* = 0.15, *p* < 0.001, 95% CI [0.13, 0.18] the effect size has increased. At the + 1SD the relationship was still significant *b* = 0.20, *p* < 0.001, 95% CI [0.16, 0.25] with the effect size still increasing. Overall, this analysis suggests that SCS moderates the relationship at all levels between ONI and EDQOL.

**Table 4:** Results of moderation analyses using ONI to predict EDQOL with self-compassion as moderators

	SCS			
	*Model statistics*			
	*R*	*R*2	*f*	*p*
	0.63	0.39	55.98	** < 0.001**
Predictors	*Predictor statistics*			
	*b*		*p*	95% CI
ONI	0.15		** < 0.001**	0.12 to .18
SCS	− 0.38		0.11	− 0.84 to 0.08
Interaction	0.076		**0.0027**	0.027 to 0.13

SCS: Self-compassion scale. Bold figures indicate significant figures

The second moderation model analysis used ONI as the independent variable, EDQOL as a dependent variable, and FFMQ and components of FFMQ as potential moderators. The results indicated that the awareness subscale of FFMQ (*p* = 0.03) acted as a moderator in the relationship between ONI and EDQOL. This has been explored with the use of simple slopes around the EDQOL mean, at the -1SD there was a significant relationship between ONI and acting with awareness (FFMQ) *b* = 0.13, *p* < 0.001, 95%CI [0.10, 0.16], while this remained significant at the EDQOL mean *b* = 0.16, *p* < 0.001, 95% CI [0.14, 0.19] the effect size has increased. At the + 1SD the relationship was still significant *b* = 0.19, *p* < 0.001, 95% CI [0.15, 0.24] with the effect size still increasing. The rest of the moderators were non-significant. The results are presented in Table 5.

**Table 5:** Results of moderation analyses using ONI to predict EDQOL with mindfulness and constructs of mindfulness as moderators

FFMQ				Describing (FFMQ)				Non-Judgement (FFMQ)			
*Model statistics*				*Model statistics*				*Model statistics*			
*R*	*R2*	*f*	*p*	*R*	*R*^2^	*f*	*p*	*R*	*R2*	*f*	*p*
0.61	0.38	50.78	< 0.001	0.62	0.38	53.10	< 0.001	0.63	0.40	56.59	< 0.001

Predictors	*Predictor statistics*			*Predictor statistics*			*Predictor statistics*		
	*b*	*p*	95% CI	*b*	*P*	95%CI	*b*	*p*	95%CI
ONI	0.15	** < 0.001**	0.13 to 0.18	0.16	** < 0.001**	0.13 to 0.18	0.15	** < 0.001**	0.12 to 0.18
FFMQ	− 0.05	**0.023**	− 0.09 to − 0.01	− 0.11	0.052	-0.22 to 0.001	− 0.18	**0.0018**	− 0.30 to − 0.07
Interaction	− 0.001	0.76	− 0.005 to 0.004	− 0.01	0.053	-0.02 to 0.0001	0.007	0.23	− 0.005 to 0.02

Observing FFMQ)				Awareness (FFMQ)				Non-Reactivity (FFMQ)			
*Model statistics*				*Model statistics*				*Model statistics*			
*R*	*R*^2^	*f*	*p*	*R*	*R*^2^	*f*	*p*	*R*	*R*^2^	*f*	*p*
0.61	0.37	49.27	< 0.001	0.62	0.38	52.90	< 0.001	0.61	0.37	50.07	< 0.001

Predictors	*Predictor statistics*			*Predictor statistics*			*Predictor statistics*		
	*b*	*p*	95% CI	*b*	*P*	95%CI	*b*	*p*	95%CI
ONI	0.16	** < 0.001**	0.13 to 0.19	0.16	** < 0.001**	0.14–0.19	0.16	** < 0.001**	0.13 to 0.19
FFMQ	0.05	0.43	− 0.07 to 0.17	− 0.07	0.25	− 0.20 to 0.05	− 0.06	0.38	− 0.19 to 0.07
Interaction	− 0.007	0.21	− 0.02 to 0.005	0.01	**0.03**	0.001–0.025	− 0.003	0.52	− 0.01 to 0.01

*FFMQ* Five Facet Mindfulness Questionnaire. Bold figures indicate significant figures

Furthermore, the third moderation model analysis used ONI as the independent variable, EDQOL as a dependent variable, and the four components of MEBS as potential moderators. The results indicated that none of the MEBS components were significant moderators in the relationship between ONI and EDQOL. However, the subscale of Hunger and Satiety was close to the significance level with *p* = 0.053. The results are presented in Table 6.

**Table 6:** Results of moderation analyses using ONI to predict EDQOL with mindful eating and constructs of mindful eating as moderators

MEBS				Hunger and Satiety (MEBS)				Distraction (MEBS)			
*Model statistics*				*Model statistics*				*Model statistics*			
*R*	*R*^2^	*f*	*p*	*R*	*R*^2^	*f*	*p*	*R*	*R*^2^	*f*	*p*
0.68	0.27	71.51	< 0.001	0.65	0.42	61.63	< 0.001	0.63	0.40	55.28	< 0.001

Predictors	*Predictor statistics*			*Predictor statistics*			*Predictor statistics*		
	*b*	*p*	95% CI	*b*	*p*	95% CI	*b*	*P*	95% CI
ONI	0.14	** < 0.001**	0.11 to 0.16	0.14	** < 0.001**	0.12–0.17	0.15	** < 0.001**	0.13 to 0.18
MEBS	− 0.08	** < 0.001**	− 0.11 to − 0.06	− 0.14	** < 0.001**	− 0.19 to − 0.08	− 0.15	**0.001**	− 0.24 to − 0.08
Interaction	− 0.002	0.09	− 0.004 to 0.0003	− 0.004	0.053	− 0.009 to 0.0001	0.001	0.80	− 0.01 to 0.01

Focused Eating (MEBS)				Awareness (MEBS)			
*Model statistics*				*Model statistics*			
*R*	*R*^2^	*f*	*p*	*R*	*R*^2^	*f*	*p*
0.63	0.39	55.24	< 0.001	0.66	0.43	64.50	< 0.001

Predictors	*Predictor statistics*			*Predictor statistics*		
	*b*	*p*	95% CI	*b*	*p*	95% CI
ONI	0.15	** < 0.001**	0.12 to 0.17	0.15	** < 0.001**	0.12 to 0.17
MEBS	− 0.11	**0.002**	− 0.17 to − 0.04	− 0.22	** < 0.001**	− 0.30 to − 0.14
Interaction	− 0.002	0.36	− 0.007 to 0.003	0.002	0.62	− 0.01 to 0.01

*MEBS* Mindful Eating Behaviour Scale. Bold figures indicate significant figures

## Discussion

The primary aim of the current study was to explore the associations between ON, mindfulness, mindful eating, self-compassion and eating disorder QoL, as well as the potential moderation of self-compassion, mindfulness, and mindful eating. Previous research into orthorexia and mindfulness has stated that there was a negative correlation between the two constructs [84]. The findings in the present study have confirmed the same relationship between orthorexia and mindfulness, and are in line with eating behaviours research and mindfulness, as mindfulness is associated with healthier eating [8, 29, 52–54, 56–59] and protective values against the development of disordered eating [67]. Two of the subscales were related negatively to orthorexia, non-judgement and acting with awareness. Research showed that individuals with orthorexic behaviours display high levels of distress, self-judgement, and self-punishment when dietary violations occur [11, 48]. However, individuals with high orthorexic tendencies displayed low levels of acting with awareness. Such a relationship with acting with awareness goes against findings in the orthorexia literature, as research suggested that such individuals engage in obsessions with nutrition, where their entire focus is on the preparation of food and ensuring the quality of food before consumption [48], which may or may not be a different description of being aware that needs further exploration. Findings may be relevant to recent literature dictating a separation of decision-making around food from mindful eating behaviours [50], and food preparation would certainly not relate to decision-making in the present moment as described in both mindfulness and mindful eating.

Self-compassion and mindful eating have also been investigated in the present study. A previous study by Kalika et al. [43] showed a negative relationship between self-compassion and ON, but no relationship between mindful eating and ON. The finding in the present study regarding self-compassion has been in accordance with past research [43]. However, mindful eating has also been significant in the present study, which contradicts the findings by Kalika et al. [43]. Thorne et al. [89] also investigated the role of mindful eating on ON and the findings showed that there was a negative relationship between some of the constructs of mindful eating, indicative of proposals for the separation of decision-making for mindful eating from the eating behaviour that occurs as a result of mindful eating guidance. Previous research has shown that self-compassion is associated with a variety of positive eating behaviours, individuals with higher levels of self-compassion tend to have lower levels of disordered eating, as well as more intuitive eating that relies on satiety cues and lower dietary restraint [82]. Furthermore, high self-compassion has been linked to more mindful eating, lower disordered eating, and lower BMI [53, 54, 88]. In addition, previous findings also demonstrated a clear link between self-compassion and mindful eating [31, 46], which was replicated by the findings in the current study.

The present study looked at the constructs of mindful eating and there were three significant relationships with orthorexia. The subscales of *eating with awareness, focused eating and hunger and satiety* were all negatively associated with ON. As previously mentioned, only three other studies looked at mindful eating, Kalika et al. [43] found no associations between ON and mindful eating whereas Thorne et al. [89] found negative relationships between ON, hunger and satiety, eating with awareness and eating without distractions. This study replicated the findings of Thorne et al. [89], however, in the current study eating without distractions was non-significant. A reason for contradicting findings of Kalika et al. [43] was that they investigated vegan-only population, therefore, this could explain the variation in the results in regard to mindful eating. It is interesting that ON has been negatively associated with eating with awareness as individuals with orthorexic tendencies focus on the quality of their food [48]. Hunger and satiety subscale was negatively associated with ON, suggesting that individuals high with ON respond to external food cues, like other EDs such as BED [64, 65] and do not rely on hunger and satiety. Findings related to the focused eating subscale are interesting as studies on orthorexic tendencies suggest that those with high orthorexic tendencies spend a significant amount of time preparing their meals and researching (Koven & Arby, 2015) thus higher focus on the eating-related behaviours, however, the present study suggests that those with high orthorexic tendencies in fact have low focused eating. This confirms findings on restraint eaters and attention bias, where research suggests that individuals with restraint pathology have an increased attention bias for food cues, which results in increased food cravings and food intake [66]. These findings are very interesting, however, caution needs to be taken when interpreting the findings. Keyte et al. [46] have highlighted possible limitations of the MEBS scale that this scale focuses more on the attentive rather than mindful eating aspect of behaviour, which are two separate concepts of eating literature altogether. Furthermore, Mantzios [50] has suggested that hunger and satiety may in fact not relate to mindful eating, but to the decision-making prior to engaging in eating. While there are several limitations that have been highlighted in measuring mindful eating, and the choice of using the MEBS was the best choice available, future research should aim to develop and explore mindful eating through more valid and appropriate measures.

This is the first study that investigated the eating disorder quality of life in relation to ON. Past research has demonstrated that individuals with eating disorders display poor quality of life (Agh et al., 2016; [21, 41, 95], this is demonstrated in the present study as individuals with higher scores on ONI displayed lower levels of quality of life. The findings indicate that higher scores on ONI have an impact on all the subscales of the EDQOL such as the psychological, physical/cognitive, financial and work/school aspects, therefore, demonstrating that orthorexia could significantly impact individuals’ quality of life, affecting physical, psychological, financial and work aspects. This highlights that higher orthorexic tendencies have a significant impact on individuals’ quality of life just like other ED that are presented in the DSM-5 such as AN, BN and BED (e.g., [6, 41, 61]. Exploring QoL is important, especially in association with ON, as there are no known interventions for orthorexia.

The current study has also utilised the use of ONI to assess the severity of ON in the current sample. Most of the research into ON has used scales such as Dusseldorf Orthorexia Scale and ORTHO-15 (e.g., [7, 86]. Only two studies to date have used ONI [44, 72] which showed a similar mean score as the present study. The current study showed a mean of 36.53 whereas Kaye et al., (2021) showed a mean of 39.03 in their female sample and Oberle et al., [72] showed a mean of 41.13 in their mixed sample. The present study had the lowest mean score compared to the other two studies, which could be a result of using specific populations such as nutrition and psychology students [72]

In addition, several moderation analyses were conducted between orthorexia and eating disorder quality of life with moderators being mindfulness, self-compassion and mindful eating. The current study found significant moderators to be self-compassion and the awareness aspect of mindfulness. The findings in the present study showed that self-compassion is a moderator at all levels with higher levels of self-compassion having a higher moderating effect on the relationship between ON and EDQOL. This suggests that higher levels of self-compassion in fact strengthen the relationship between orthorexia and quality of life. This is an unexpected finding as the associations in the present study showed that there was a negative relationship between self-compassion and orthorexia and quality of life. Taking into account what is known about self-compassion and the associations in the present study self-compassion should have weakened the relationship between orthorexia and quality of life. Past research has demonstrated that self-compassion in fact is interlinked with better quality of life in individuals who displayed anxious and depressive symptoms [91]. The findings of the present study go against this suggesting that individuals with high orthorexic tendencies and high self-compassion will demonstrate a worse quality of life. A reason for this could be that individuals with high self-compassion believe that engaging in healthy eating rituals and physical activity are means of improving their optimum health and a form of self-care [30, 49, 51], however, orthorexic tendencies have been shown to impact an individual in a social and psychological way resulting in lower quality of life. Another significant moderator was the awareness facet of mindfulness, acting with awareness suggesting that the individual is focusing all the attention on a current activity (Brown et al., 2015 such as food preparation or researching the organic and pure produce. Again, this goes against the associations presented in the present study as there were negative relationships between awareness and orthorexia and quality of life. Research has shown that individuals with high orthorexic tendencies often obsess about their eating behaviours and regimen [11]. Acting with awareness was also a moderator at all levels with higher levels of awareness having a higher effect on the relationship between ON and EDQOL. The findings of the present study go against our understanding of utilising self-compassion and mindfulness concepts in populations with disordered eating, mindfulness-based interventions have been shown to be effective as a treatment for eating disorders (e.g., [92]. Research shows that self-compassion and mindfulness promote healthy eating (e.g., [4, 63, 92], which could explain why self-compassion and awareness were moderators as individuals with high orthorexic tendencies believe that they are engaging in healthy eating behaviours and might be utilising these concepts as forms of self-care and promoting optimum health.

## Limitations

A clear limitation of this study is the female-only sample, therefore the findings cannot be generalised to male populations. Gender differences are consistently observed in eating pathology [9, 85] and some studies into ON has shown that symptomology has been greater in men than women (e.g., [34]. However, findings into ON research are inconsistent as other studies in fact show that the symptomology is greater in women (e.g., [26] and other studies suggest that there are no gender differences (e.g., [17], Dunn et al., 2017; [38]. Therefore, future research should focus on equal male recruitment and conducting studies with male-only populations as there is a lack of literature across the field.

Furthermore, the present study has utilised the ONI to measure the ON severity in this sample. This is a new measure of ON that has only been used in two previous studies [44, 72], even though this measure assesses physical impairments and emotional distress. Caution should be taken as the ONI should be used as a measure to assess the risk of ON development, rather than a diagnostic tool. Additionally, previous research that supports and contradicts the findings of the present study have used different measures of ON such as DOS and TOS (Kalika et al., 2021, Straus, 2020). For example, Kalika et al. (2021) showed that mindful eating was not related to ON whereas the present study showed a negative relationship between the two constructs. Therefore, future research should utilise the ONI as a measure of ON to further investigate concepts of mindfulness and self-compassion.

Another limitation is that only associations of EDQOL can be made to ONI due to the sample not having been diagnosed with ON. The conclusions drawn from this measure can only be that those with higher ONI had poorer ED quality of life, and conversely, those with lower or less ON symptoms did not have better QOL, but that their eating or weight did not affect their quality of life. Therefore, future research using the EDQOL should use a sample that consists of individuals meeting the recent diagnostic criteria for ON.

## Future directions

There is a need for qualitative research to be conducted on the ON populations. Exploring qualitative research will allow a further understanding of how self-compassion, mindfulness and mindful eating are utilised in this population. There is limited literature available that has explored ON qualitatively (e.g., [19, 90, 93], and this could potentially lead to the official classification of ON, especially since the quality of life is an important aspect as individuals with a classified eating disorder display lower levels of quality of life (Agh et al., 2016, [21, 41, 61, 95]. There is also the question about the pleasure of eating, as suggested by Egan and Mantzios [31] in their qualitative study, where individuals could engage in unhealthy eating behaviours due to utilising the concept of self-kindness and treating themselves with unhealthy foods, which in turn, could lead to weight gain. Egan and Mantzios [31] further explained that social aspects rather than actual food are derivative of individuals finding pleasure in eating, and it would be beneficial to see whether that trend also occurs in orthorexic populations when past research has indicated that they usually avoid social situations [15], Sfeir et al., 2021).

Future research should investigate self-compassion, mindfulness, and orthorexia nervosa using an experimental approach with mindfulness-based and self-compassion-based interventions to help determine their effectiveness. As orthorexia research advances, developing interventions for this disorder will become increasingly important.

## Conclusion

The present research offers novel insight into ON, mindfulness, self-compassion, and mindful eating. This study has demonstrated that there is a negative association between mindfulness and orthorexia, and this relationship was also replicated with self-compassion and two subscales of mindful eating. The potential benefits are apparent as these constructs could offer an effective tool in treating orthorexia in female populations. Furthermore, the present study is the first study that has explored eating disordered quality of life and orthorexia, potentially adding to the discussion of classification and addition to clinical disordered eating protocols.

### What is already known on this subject?

Previous studies provided evidence that orthorexic eating behaviours have a relationship between mindfulness, mindful eating and self-compassion. However, the findings in relation to these constructs have been mixed, proposing the need for further research.

### What does this study add?

This study explored orthorexic behaviours by using the new Orthorexia Nervosa Inventory (ONI) and its relationship to self-compassion, mindfulness and mindful eating, The results supported previous findings that indicated that there was a negative relationship between orthorexia and mindfulness, self-compassion and mindful eating. Furthermore, the present study found a positive relationship between lower quality of life and orthorexia. Moreover, the findings indicated that self-compassion and the awareness facet of the mindfulness questionnaire moderated the relationship between Orthorexia Nervosa and Quality of Life.

## Data Availability

The datasets generated during and/or analysed during the current study are available from the corresponding author upon reasonable request.
